# Environmentally induced, occupational diseases with emphasis on chronic kidney disease of multifactorial origin affecting tropical countries

**DOI:** 10.1186/s40557-016-0119-y

**Published:** 2016-08-05

**Authors:** Shehani A. Wimalawansa, Sunil J. Wimalawansa

**Affiliations:** 1 School of Business, Rutgers University, New Brunswick, NJ USA; 2 Cardio Metabolic Institute, Somerset, NJ USA

**Keywords:** Agrochemicals, Agriculture, Agribusiness, Contamination, Human diseases, Kidney disease, Occupational hazards, Premature death, Prevention, Policies, Pollution, Water

## Abstract

**Background:**

Environmentally induced, occupational diseases are increasing worldwide, especially in rural agricultural communities. Poverty-associated malnutrition, environmental hazards and pollution, and lack of access to clean water, safe sanitation, and modern healthcare facilities are often associated with these chronic illnesses.

**Method:**

The authors systematically reviewed occupational public health issues that have been related to the environment. General interpretations of results were included as per the guidelines of the Preferred Reporting Items for Systematic Reviews and Meta-Analyses. Pertinent publications from research databases were reviewed on (A) the risk–benefits, (B) the prevalence of risk factors for various diseases, (C) the benefits of not ignoring the risk factors (i.e., broader evidence), and (D) the risks, effects, and outcomes of different types of interventions. The authors used chronic kidney disease of multifactorial origin (CKDmfo) as an example to explore the theme. Emphasis was given to the regions with emerging economies and developing countries located in the vicinity of the equator.

**Findings:**

Geographical, socio-economic and aetiological similarities exist for many chronic non-communicable diseases that are affecting tropical countries around the equator. The authors identified manufacturing, mining, and agriculture as the biggest polluters of the environment. In addition, deforestation and associated soil erosion, overuse of agrochemicals, and irresponsible factory discharge (e.g., chemicals and paint, from rubber and textile factories, etc.), all contribute to pollution. To decrease the escalating incidences of environmentally induced diseases, governments should work proactively to protect the environment, especially watersheds, and take steps to minimise harmful occupational exposures and strictly enforce environmental regulations.

**Conclusion:**

Creating public awareness of environmental issues and their relationship to public health is essential. This includes regular monitoring and periodic publication of the quality of water, air and soil; preventing deforestation and man-made soil erosion, increasing forest and ground cover, preventing occupational injuries, judicious and safe use of agrochemicals, sustainable agriculture and development programs, and implementing legislation to protect and conserve water heriage and the environment. These actions are essential both for a healthier environment and for the health of the people who live in that environment. Such measures would also decrease public health threats from such, including global-warming-related erratic environmental changes and the occurrence and the spread of non-communicable diseases, such as CKDmfo.

## Background

The anthropogenic impact on fauna, flora [1], and the biophysical (soil, air, water) environment [2] is well known. In addition, anthropogenic effects on resources, food and energy production have also been studied [3]. It is also well documented that, consumption and even bathing in polluted water could leads to many human diseases and premature deaths. To maintain clean water, watersheds and water bodies must be protected and properly maintained [4]. Officials who have authority over the environment need to understand the ecosystems and processes operating in and around watersheds and how they are affected by anthropogenic activities.

Watersheds must be protected from hydrological, chemical, and biological pollution, and should be provided with an integrated support system for the maintenance of the soil–water environment and the restoration of aquatic systems [5]. Environmental protection agencies (EPAs) need to draft and implement laws requiring analyses of ecosystem functions, ecological health indicators, future public health and environmental risks, and assessments of the health of rivers, lakes, estuaries, coasts, and wetlands [5].

A major worldwide concern is that fresh water and food are being contaminated secondary to expanding unsustainable development associated anthropogenic activities, as well as agriculture and agrochemical overuse. Matters are made worse by the human and industrial waste and agricultural runoff, all of which cause pollution, while some may cause serious diseases. Water pollution from microbes causes identifiable diarrheal illnesses. Whereas, contaminations due to chemicals and toxins are insidious and may cause premature deaths. 

Planners and authorities need to take into account, point (e.g., factories and industrial sources) [6, 7] and non-point sources of water pollution (e.g., runoff from agrochemicals and livestock, discharge from municipalities and mining) [8–10]. With the Green Revolution, non-point source pollution has emerged as the major concern.

### Water sources and quality

Drinking water comes from surface and ground sources, including rivers, reservoirs, lakes, streams, springs, and underground aquifers [4]. When rainwater and surface water travel over the ground or underground [11, 12], they collect contaminants, including naturally occurring substances, such as salts and minerals, and manmade substances such as agrochemicals, agricultural and factory waste, and animal and human waste [4, 13]. Consequently, all sources of fresh water contains a certain amount of contaminants. Environmental protection agencies and regulatory standards institutes in each country provide local quality standards for water and, regulate the amounts of contaminants allowable in the water provided by public (e.g., municipalities) and private systems.

Water quality fluctuates continuously depending on the season; whether it is the farming season, climate conditions such as drought, rain, floods, as well as agricultural and mining practices, and from industrial discharges, but can also fluctuates even within a day. Because water quality changes occur rapidly and can differs markedly even within a local area. Thus, broader conclusions cannot be made from sporadic or random testing of water samples from the field; such data are unreliable and the conclusions made are usually misleading. Therefore, one should not make generalized or sweeping conclusions about an entire region or the status of a disease by extrapolating analytical data from few sets of water samples collected at a few times of the day or infrequently over the year.

### Surface and ground water quality

Similarly, the ground water table fluctuates depending on the climate (markedly vary between rainfall and drought periods) and secondary to over-utilization of ground water via agro-tube wells. Monsoon rain and intermittent storms may result in temporary replenishment of the ground water table and wells but the associated flooding causes contamination of most water sources.

Meanwhile, water retention in catchments depends on the availability of forest and ground cover, soil conditions and erosion, and the rate of rainfall; adverse conditions of any of these can directly affect the environment and indirectly aggravate environmentally-related diseases, such as CKDmfo as well as vector-borne diseases. In addition to the ongoing climatic changes, modification of any of the above-mentioned resources can adversely affect the water cycle and thus, the human health.

Over the past five decades, short-sighted, over-zealous, environmentally detrimental programs have been implemented in many developing countries to achieve for short-term gain. These have adversely affected water quality, soil, air, fauna and flora, and human and animal health. Contributing factors include the overuse of both synthetic and organic agrochemicals, deforestation and associated soil erosion, diversion of streams and rivers, nitrogen and phosphorus-related eutrophication of reservoirs, and the proliferation of toxic human waste.

**Fig. 1:**
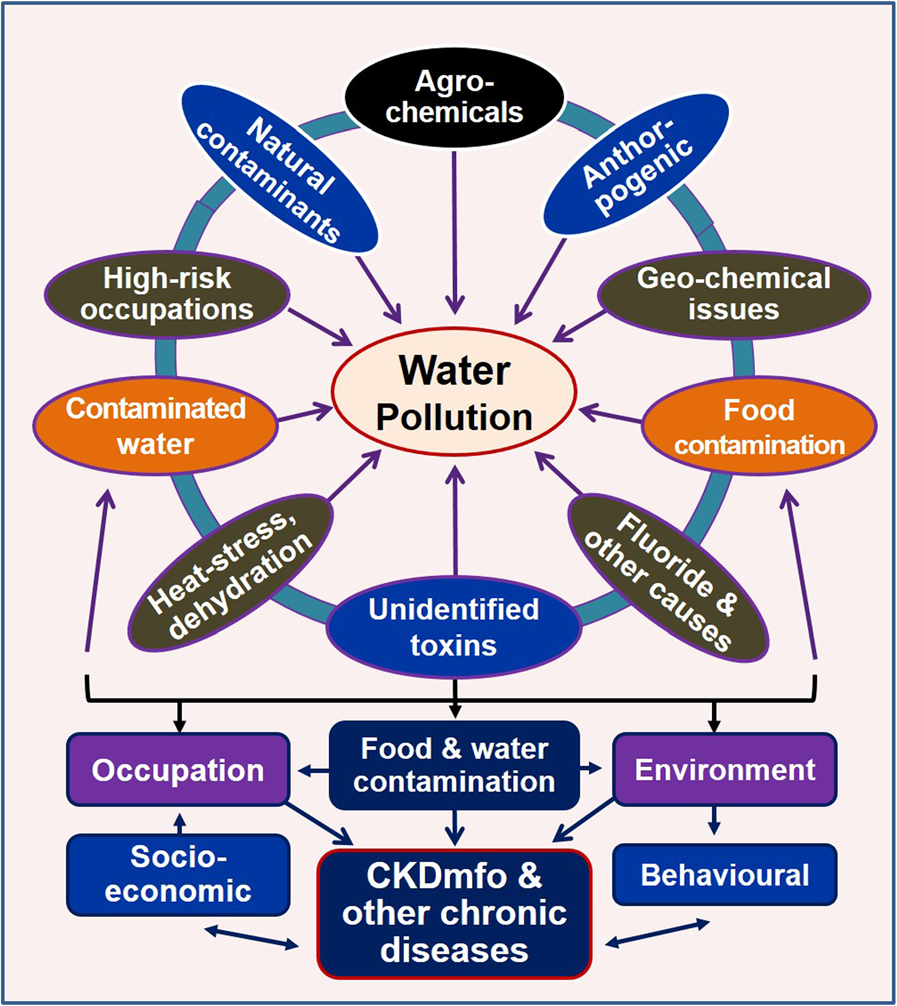
The common factors contributing to water pollution. Also shown are the interactions and interconnectedness of the factors leading to water pollution and adverse human health and the role of socioeconomics, behaviour, and occupational and environmental components in the genesis of CKDmfo

This review focuses on common sources of water pollution and their effects on human health with special reference to chronic kidney disease of multifactorial origin (CKDmfo), also known as *chronic kidney disease of uncertain aetiology/origin* (CKDu, CKDuo). Figure 1 illustrates the common sources and factors that lead to water pollution.

### Modes of water pollution

Reservoirs receive water from rainfall, drainage from streams and rivers, and runoff from upland areas and watersheds. Most rivers originate at higher elevations, where there is more rainfall and greater forest cover. Deforestation leads to less absorption of water and in turn filtration of contaminants. Contaminants from households, farms (animal waste and agrochemicals), and industries also enter streams and reservoirs located hundreds of kilometres downstream from the original contamination. In addition, water from many rivers are diverted to reservoirs for agricultural purposes and/or for power generation.

Water pollutants include household waste, fertilisers, pesticides, insecticides, heavy metals from from phosphate fertiliser, discarded batteries and workshops, and chemical waste from rubber and apparel production, vehicle repair shops, and other industries that use chemicals [14–19]. For many substances, permitted upper limits (thresholds) in drinking water have been established [20] by the World Health Organization, by EPAs, and internal standards institutes, such as the Standards Institute Sri Lanka [21–23]. These organisations have produced safe-upper limits (maximum allowable limits; MAL) many components, including for heavy metals, fluoride, agrochemicals, and total dissolved substances [24].

In most countries, the non-point sources of plant nutrient pollution through agricultural practices are main contributors to water pollution and nutrient-related eutrophication [25–27]. Although water contamination with raw sewage is a serious issue, laws regulating the treatment and discharge of sewage have led to a dramatic reduction in the contamination of watersheds and surrounding ecosystems from this source [28].

However, the implementation of regulations for agrochemicals and animal waste lag behind these efforts in most agricultural communities, particularly in developing countries [25, 26, 29]. The participation of all stakeholders, including the public, private industry, technocrats, legislators, government agencies, and environmental organizations [30] is essential for protecting and cleaning up water and environmental contaminations and related environmental bio-hazards [25, 26, 31].

Muddy water is contaminated water; mostly secondary to deforestation and agriculture-related upland soil erosion. Displaced silt and clay from upland streams increase the sediment load in rivers and downstream reservoirs. Silting necessitates dredging, which further contaminate water, thus, negatively affecting fauna and flora and disrupting their life-cycles, and endangering the food chain. Such would also hinder transportation, navigation, and interferes with recreational activities.

A turbid or excessively muddy waters also harms aquatic organisms. In addition to decreasing the dissolved oxygen content and the sunlight reaching on millions of living organisms, clay particles injure the gills of fish and adversely affect their spawning and survival. Likewise, muddy water also disrupts photosynthetic activities and puts the aquatic ecological balance into disarray.

### Chronic kidney disease of multi-factorial origin (CKDmfo)

Atypical chronic kidney diseases started to appear in the early 1960s in the Balkan regions [32–35] and later appeared in several other equatorial countries, including in Central America [36–42]. In addition to been a fatal disease, CKDmfo is a major socio-economic issue and a growing environmental and occupational health problem across South Asia, and Mesoamerica. It is an example of an environmentally induced, occupational exposure disease.

CKDmfo has been increasingly manifesting in equatorial (tropical) countries during the past four decades. The disease is found primarily in the dry-zonal agricultural societies of economically poor regions closer to the equator.

Climate change, and consumption of food or water contaminated with heavy metals, fluoride, and toxins cause various non-communicable diseases and illnesses leading to protracted suffering and death. In addition to causing prolonged, extreme droughts and flooding, climate change also causes excessive heat exposure [43], dehydration, heat exhaustion, heat stroke, renal stone formation, and exacerbation of pre-existing chronic disease, including kidney failure (heat-shock nephropathy).

Typically, CKDmfo affects low-income agricultural workers, predominantly males, aged 30 to 60 years, living in low-lying, flat, tropical dry-zonal areas. The disease is not associated with common causes of chronic kidney failure, such as diabetes or hypertension, and is mostly asymptomatic until kidney function is moderately impaired [44–47].

Sri Lanka has been afflicted with an epidemic of CKDmfo for more than two decades; the disease has caused the deaths of thousands of people, mostly middle-aged, male farmers [46, 48, 49]. More people have succumbed to CKDmfo than died during the 2004 tsunami in Sri Lanka [50]. Although a single causative factor has not determined, data suggest that certain levels of unhealthy exposures to unhealthy environment (a contaminated soil–water system) are likely contributing to this disease [51].

Sri Lanka reports the highest incidence of CKDmfo in South Asia, with around 180,000 people affected and approximately 50,000 reported deaths since 1992 [47, 50, 52–54]. Effective early diagnosis and preventive, and treatment strategies are still lacking [52, 55]. Likewise, environmentally friendly and maintainable agricultural practices and food security are needed, as is the enhancement of rural livelihoods [4, 53].

Depending on the studies reported globally, the correlations described between environmental pollutants and CKD of unknown aetiology (CKDu; CKDmfo) vary greatly. Reported pollutants in the global arena include arsenic in subterranean water tables in India [42], aristolochic acid in the Balkans and in certain parts in China [32, 34, 35], heavy metal exposure in Central America and South America [38, 39, 56], and a combination of various chemicals in Egypt [41] and in Sri Lanka [52]. In spite of the temporal associations and justifiable speculations, the use of agrochemicals has not yet scientifically proven to be linked to causation of CKDu/CKDmfo.

Although contributory, none of the affected countries to-date has identified agrochemicals as the sole cause of this deadly disease [52]. Therefore, labelling the disorder with such names as “agricultural nephropathy” or “agrochemical nephropathy” is misleading and only further delays the identification of the true cause(s).

Nevertheless, considering the temporal association of the “introduction” of agrochemicals in early 1960s', the indiscriminate and overuse of agrochemicals, flat terrain, with poor drainage in the affected regions, scarcity of potable water, and consumption of water that is contaminated with several chemical components, agrochemicals are likely to be one of the agents contributing to CKDmfo [57].

Although no causality has been established for the CKDmfo in Sri Lanka, it has been established that exposure to combinations of various nephrotoxins and chemicals over a long period, even at lower exposure levels (i.e., lower than the MAL), can lead to bio-accumulationof harmful agents, which lends further support the idea of multifactoril origin and for acausal relationship [33].

In Sri Lanka, the disease first appeared in the early -1990s and affected mostly the North Central Province(NCP). Several recent studies failed to detect higher than the maximum allowable limits (MAL) of any of the postulated components of heavy metals, fluoride, salinity, or agrochemicals in the soil, water, or food of this region. Consequently, none of these items though hypothesized as a single cause, have been linked as the cause of CKDmfo [54, 58–60]. Moreover, none of the compounds measured (except water fluoride contents in certain villages) are higher than those deemed safe by the World Health Organization and the U.S. Environmental Protection Agency [59].

Nevertheless, the additive or synergistic effects of a combination of factors, including consumption of contaminated water, even with these components at lower than the maximum allowed exposure levels when associated with climate change, malnutrition, and/or harmful behaviours could cause this major epidemic. For example, current data suggest that the consumption of water polluted with certain nephrotoxins associated with unsustainable development practices together with exposure to the consequences of climate change and possible heat-stress nephropathy and so forth, can cause chronic renal tubular damage [43, 47].

Victims are primarily from dry zonal regions in the affected, predominantly agricultural countries and mostly consume shallow well water that is thought to be contaminated. However, approximately 15 % of the CKDmfo-affected families rely on drinking water from other than shallow-wells: from deep-tube wells, canals, and reservoirs. Therefore, it is unwise to assume a single source or type of water, or a presumed/unknown contamination as the cause of CKDmfo [46, 49, 61, 62].

### Agrochemicals and CKDmfo

Published data support a plausible, temporal correlation between exposure to agrochemicals and increased risk of chronic diseases, such as certain cancers, neurodegenerative disorders, cardiovascular diseases, asthma, mitochondrial dysfunction, oxidative stress, birth defects, endocrine disruptions, and reproductive disorders [63]. Therefore, from a risk perspective, minimising pollution from such chemicals should be given a high priority irrespective of whether it is linked to CKDmfo or not.

Nevertheless, increased agricultural output essential to feed the world’s ever-increasing population, which is greatly relies on agrochemicals, thus, the use of agrochemicals has become standard practice worldwide. Globally, less than 8 % of food cultivations use organic methods. Of this type of cultivation, 80 % is used for animal feeding (pasture) to produce organic meat.

Sri Lanka and in all other CKDu-affected countries (and regions), these chemicals are extensively used throughout the country, but only people living in certain geographical areas only are experiencing this disease. This suggests the contributions of other factors in the genesis of CKDmfo.

Factors such as climate change, prolonged dry seasons, behavioural issues, malnutrition, illegally brewed alcohol, medication abuse, and microbial-induced toxins all remain as possible contributing factors. With reference to the hill country in Sri Lanka, agrochemicals are used at rates several times greater (and 5 to 10 fold higher amounts than recommended by the department of agriculture) than in the region that is most affected with CKDmfo: the NCP. Thus, the agrochemical-based hypothesis cannot explain why only certain geographically demarcated areas are affected, whereas people in other regions (sometimes adjoining villages and regions) exposed to the same nephrotoxins are not affected.

Other than the nitrates and phosphates found in water, no meaningful quantities of pesticides (insecticides, weedicides, or nematocides) have been reported in food items by any analytical study published to date. Similarly, no pesticide residues have been detected in cadaveric tissue samples. The latter is important with reference to countries that have banned certain pesticides over a decade or so.

If trace amounts of such substances are detected in blood, urine, or tissue samples, as some have 'claimed' (stating, unpublished data), that finding indicates the (a) illegal importation of banned pesticides into the country or (b) imported food has been contaminated with such substances. In addition, no data are available regarding the hypothetical claim or detection of 'pesticide–metal' complexes in renal or any other tissues.

**Fig. 2:**
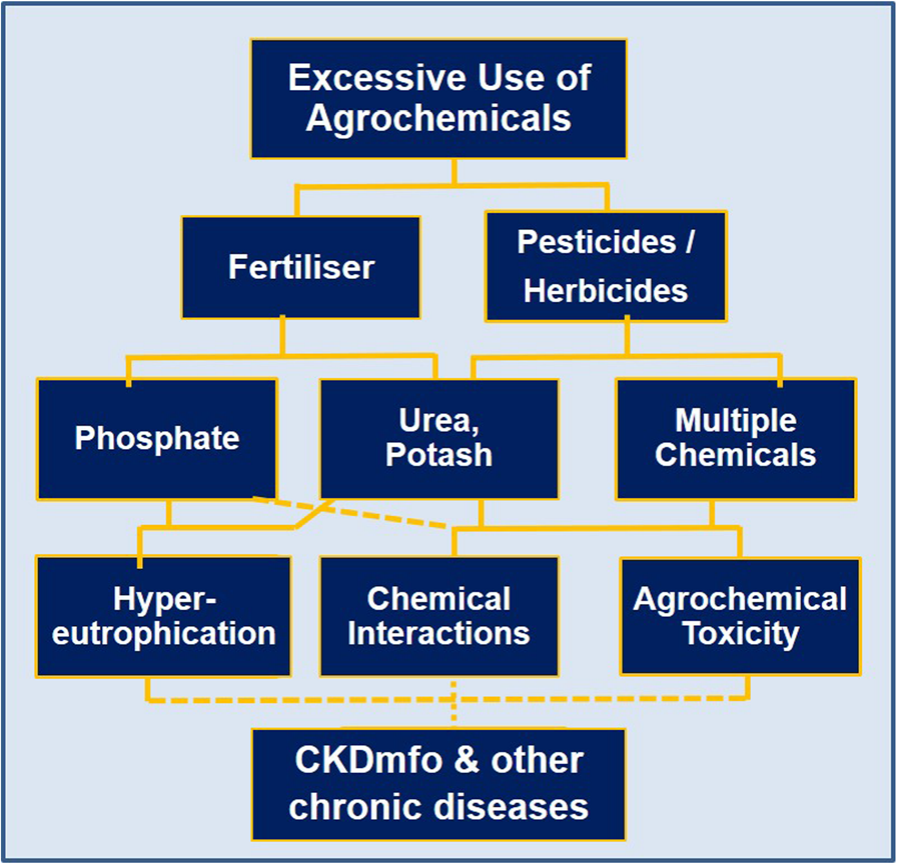
Hypothetical interactions and interrelationships of excessive and irresponsible use of agrochemicals and the development of CKDmfo, a hypothesis that has not been fully tested or proven

If traces of heavy metals or pesticides are detected in urine or tissue samples, that does not prove the particular substance is causally related to the failure of a given organ. When an organ such as the brain, liver, or kidney begins to fail, it loses internal dynamics and the ability to control what is retained and excreted. Thus, the finding of comparatively high levels of arsenic in urine or of traces of a heavy metal or pesticide residue in renal tissue does not mean that these substances are causing disease. Instead, it is a consequence of the disease. Figure 2 illustrates one such, agrochemical-based hypothesis as the cause of CKDmfo.

### Contamination of water and food via herbicides and pesticides

To increase the output, farm crops such as potatoes and vegetables require regular application of fertilisers (i.e., organic or agrochemicals) and pesticides. When pesticides are applied in higher than the recommended quantities, insects gradually become resistant to them, so more pesticides are needed to control pests, leading to a cycle of escalating chemical use [51].

Tea estates apply herbicides such as glyphosate three times a year, but none of the plantation workers are affected with CKDmfo. Similarly, workers who regularly use the largest quantities of pesticides and fertilisers on per hectare basis [e.g., triple super-phosphate (TSP), pesticides, glyphosate, etc.], such as those in the Hill Country region, are not affected with CKDmfo. Whereas, the prevalence of the disease among adults are over 10 % in some of the CKDmfo affected villages in the NCP.

### Do agrochemicals or bad practices contribute to the CKDmfo?

Using the correct amount of agrochemicals (whether in-organic or organic; as recommended by the department of agriculture) at the correct time of the farming season, would increase the crop output, while minimising environmental harm [64–66]. On the other hand, the excessive use of agrochemicals contributes not only to hyper-eutrophication of water in reservoirs but also to contamination of the soil and water with heavy metals and other naturally occurring chemical constituents in fertiliser [4, 66].

In most of the affected countries, including Sri Lanka, many of these problems can be traced back to the elimination or "reduced" communication between village-level agricultural officials and farmers that began in the late 1970s [4]; i.e, after the abolition of agricultural extension services by the government. Removal of this important advisory service to farmers led to several negative outcomes, including fertiliser overuse.

In addition, most farmers in developing countries who use agrochemicals do not use protective gear and thus frequently are exposed to higher concentrations of toxic chemicals and are at a higher occupational risk for experiencing chemical-induced acute and chronic diseases. These observations are applicable to almost all farmers in Sri Lanka and other CKDu-affected countries. Nevertheless, the excessive or careless use of agrochemicals do not explain the sporadic geographical distribution of this disease.

### The role of chemical and organic fertilisers

Since the advent of the Green Revolution, the use of organic and natural fertilisers has decreased and the use of chemical fertilisers has increased. Currently, more than 95 % of farmlands worldwide are cultivated using chemical fertilisers. Organic fertilisers include manure, crop residue, tree leaves, animal waste, and compost [21], all of which can be locally produced, with much of the nutrients being derived from natural sources.

However, the amounts of organic fertiliser generated have not changed significantly over time, with the exception of rice straw and wheat husks, which have increased because of the use of harvesting machines in commercial farms that return the straw back to the fields. Nevertheless, the quantities of organic fertiliser needed to provide the nutrients for growing plants (e.g., kilograms per hectare) are orders of magnitude higher than the amounts of chemical fertiliser needed. Thus, such increase from the current usage in commercial farming is impractical.

Even if the use of organic fertilisers were to be maximised, these would be only enough for less than 10 % of farming needs. On the plus side, in industrialised countries, the use of organic fertilisers, manure, and animal dung has decreased the instance of hyper-eutrophication of water bodies [22]; whilst the reverse is true in developing countries. 

With appropriate use, organic fertilisers have no adverse environmental issues [64, 65]. However, since, many types of organic fertilisers bio-accumulate toxins and heavy metals, with time, the excessive use of organic fertilisers could unintentionally introduce significant amounts of these toxic components into farming lands.

Four decades ago, soil in the rice fields in southeast Asian countries, including Sri Lanka, (particularly in the midcountry, wet zone areas of central province [67], later colonised as the NCP) were deficient in phosphorus, but full of fish and other living beings. The latter is non-exiting now. Phosphorus deficiency was one of the reasons for the introduction of Triple Super Phosphate (TSP) in agriculture, which led to marked increase in paddy-yield.

The availability of cultivation loans, strong extension services, good paddy prices, and most importantly, the large fertiliser subsidies helped not only increasing the paddy output but also eradicating the phosphate deficiency though the use of TSP and Single Super Phosphate (SSP).

However, during the past three decades, potato and vegetable growers in the Hill Country regions have began to add excess phosphate fertiliser to their farm lands [51]. This led to phosphate build-up in soil beyond the critical threshold, and excess phosphate has begun to leach out of farmlands, ending up in reservoirs located hundreds of kilometres away [68, 69].

**Fig. 3:**
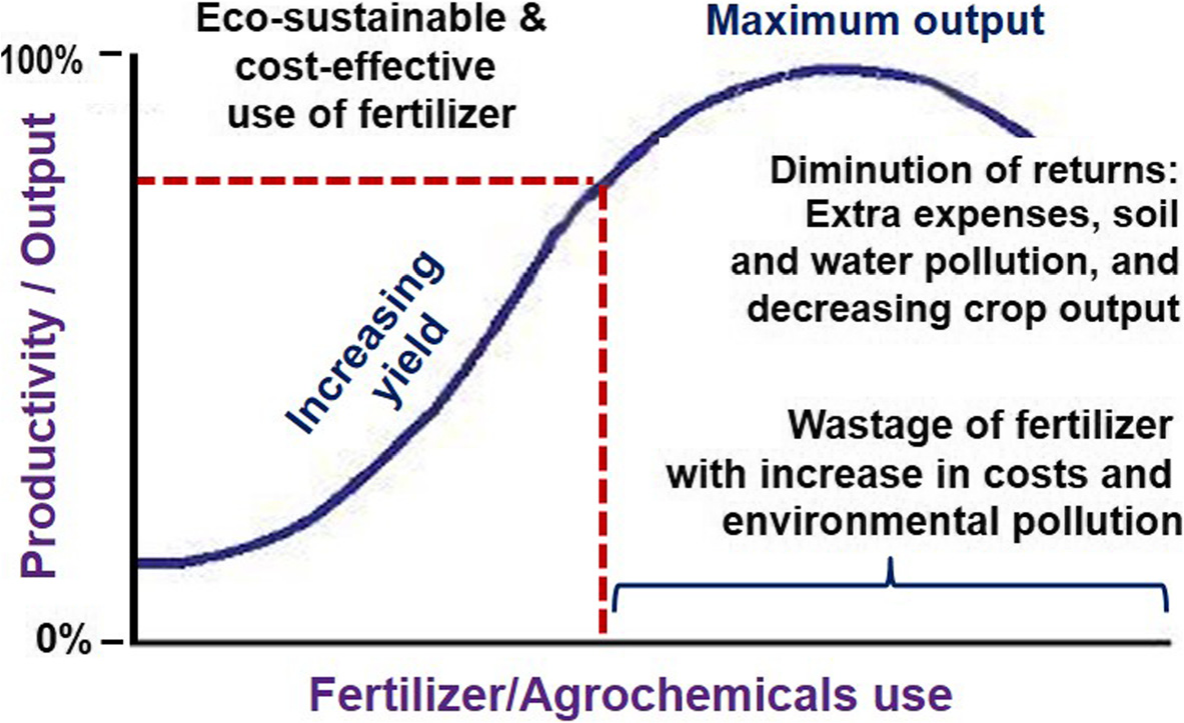
An example of productivity, showing the yield output curve in response to the increasing use of fertiliser in rice, potato, and vegetable cultivations. Crop output increases with increasing amounts of right fertiliser mixture, but at a certain point [MC = M × R], it starts to plateau and may even decline. Thus, more is not always better (adapted from Wimalawansa et al., [51, 66])

In CKDmfo-affected countries, on a per-hectare basis of arable land, fertiliser usage has tripled since the late 1970s [51]. Once the soil has reached its threshold for holding fertiliser, these chemicals leach into waterways; the money spent on fertilisers is wasted and the chemicals cause environmental pollution (Fig. 3). Farmers began to use increasing amounts of pesticides to overcome pest resistance, and  in order to obtain desired higher yields [66, 70]. The result was not always the intended one; more is not always better even in agriculture (Fig. 3) [51, 66].

### Environmental pollution from heavy metals

The term *heavy metal* usually refers to chemical elements that have a high atomic number and is toxic to living beings at low concentrations. Similar to the World Health Organisation (WHO) and EPA, the Sri Lanka Standards Institute has also identified heavy metals such as arsenic, cadmium, lead, mercury, selenium, and hexavalent chromium, etc., as toxic substances [62, 63] and have stipulated the MALs of these in drinking water, fertiliser, and farm soil [23]. Table 1 lists some of the key heavy metals that are harmful if gets into the body.  However, these are used commonly in many industries and in households, and take less precautions during their use and disposals due to inadequate awareness of their dangers.

**Table 1:** Common uses and harmful effects of heavy metals [hazardous waste] on human health [23, 72]

Substance	Use	Harmful effect	MAL in potable water^a^ (mg/L)
Cadmium	Batteries, electroplating, TSP, SSP, tobacco, illegally brewed alcohol	Protein and sugar in urine; renal damage	0.005
Lead	Paint, batteries, alloys, welding, older water pipes and joints	Nervous system and brain damage, lowered IQ, liver and kidney damage	0.05
Mercury	Thermometers, fluorescent lamps and tube lights, thermostats, thermometers, medical equipment, batteries	Nervous system and brain damage, renal damage	0.001
Hexavalent (VI) chromium	Steel manufacturing, chrome plating, magnetic tapes, dyes and paints, tanning of leather, textile industry, tobacco smoke	A carcinogen, kidney damage, skin diseases	0.05
Arsenic	Insecticides, nematocides, wood preservative, alloys with copper and lead (car batteries), pesticides, volcanic ash	Cancers in lung, bladder, and skin; keratosis and dermatitis	0.05

^a^ *MAL* maximum allowable limit. *Source*: Sri Lanka Standards Institute [23]

Most people inevitably use and handle heavy metals during day-to-day activities. Most of these materials, instruments, and technologies using heavy metals are designed to improve human lifestyles, add conveniences to peoples’ lives and increase prosperity. However, the misuse of heavy metals, especially their improper handling during production cycles and disposal, can inflict a heavy toll on the environment and consequently on peoples' health [71].

### Environmental pollution with heavy metals and human diseases

Around the world, many people have contracted diseases brought about by ingesting heavy metals and inhaling fumes; the symptoms of such diseases can be subtle [72, 73]. Consequently, many go undiagnosed, with the true causes not linked to morbidity or mortality, and thus, the number of documented deaths related to heavy metal poisoning have been underestimated. The Blacksmith Institute, an environmental health group based in New York City, in 2011 estimated that the number of people with heavy-metal–related diseases in the world is, 7.2 million related to lead; 5.0 million to mercury; and 1.8 million to chromium VI [74].

Whilst fertiliser is commonly contaminated with heavy metals, these are added to pesticides such as insecticides to increase their toxicity. However, some unscrupulous manufacturers use fertiliser as a mode of disposing chemicals and heavy metals, including cadmium and lead [52]. Such contaminants at higher than acceptable limits are not rare in fertiliser consignments shipped from some industrialised countries into emerging economies and developing countries, including Sri Lanka.

Other products containing heavy metals that are imported to these counties include batteries, vehicles, fluorescent bulbs, materials and parts, paints and chemicals, medical equipment, and agrochemicals. For example, in Sri Lanka with a population of 21 million, is a substantial importer of heavy metals. Examples of imported goods containing heavy metals include an estimated, 650,000 cars, 22 million mobile phones (each with a battery and spare batteries), and millions of other household batteries and fluorescence bulbs; most of which are unfortunately not recycled.

Each middle class family purchases 20 to 30 batteries per year and disposes of them in the garbage or buries them in soil. Meanwhile, each compact fluorescent lamp/light bulb contains approximately 3 mg of mercury, while arsenic and cadmium can be found in phosphate fertilisers and pesticides [14, 75], all of which are common modes of contaminating the environment, of which the EPA has little or no control. The lack of recycling and proper disposal systems in emerging economies, including Sri Lanka, is likely to cause further escalating major chronic health issues in years to come.

Large quantities of heavy metals in developing countries are as yet not recycled, and end up as domestic and industrial waste, a portion of which eventually pollutes soil and water systems, and eventually will get in to the food chain. To counteract this impending disaster, reliable inventories must be developed that capture the amount and types of heavy metals imported, recycled, reused, and properly disposed.

To prevent anthropogenic heavy metals entering the ecosystem, an introduction of a culture of safe-recycling is essential in all developing countries and emerging economies as soon as possible. This can be based on the established principles and safe-practices used in economically advanced nations.

### Excess heavy metals in water and soil

Small quantities of heavy metals, including arsenic, cadmium, lead, and chromium, have been detected in soil samples and a few water samples in regions and countries that are affected with CKDmfo. However, the soil distribution of various heavy metals is not unique to CKDmfo-affected regions in countries with a high prevalence of this disease or other common non-communicable chronic disorders [4, 52, 53]. If levels of these contaminants consistently exceed permissible limits (e.g., MAL), threats to human health are likely [75].

Most samples analysed by different research groups recentley, in regions with a high prevalence of chronic diseases such as CKDmfo, including in Sri Lanka, show levels of heavy metals within the EPA and WHO allowable limits [42, 76, 77]. It is unclear how such low levels can affect humans across a large region like the NCP. However, because some of the chemicals, especially heavy metals, bio-accumulate during the human life-span, in the long term these can cause chronic ill health. Therefore, if left unchecked, the presence of heavy metals, even though the levels are less than the MAL, ultimately may lead to serious illnesses, including chronic occupational diseases [49].

In addition to the metallic load, the release of ionic compounds, such as cadmium, manganese, hexavalent chromium, lead, and nickel, into the water (and eventually into food) depends on chemical parameters, such as dissolved organic carbon, ionic strength, temperature, pH, particle size, the presence of protective agents such as zinc, selenium and other antioxidants, and the frequency and depth of dredging of canals and reservoirs, etc. [77–79]. It is known that the toxic effects of heavy metals are modified in the presence of other ions. Decrease of cadmium toxicity in the presence of zinc is one such example.

With reference to CKDmfo in Sri Lanka, except for fluoride, none of the other components have been detected at levels higher than MAL in water samples [52]. Considering all data, currently there is no scientific evidence to support the theory that heavy metals in water or food, water ionicity (salinity, phosphate, etc.), nitrate, or hardness of water cause CKDmfo [4, 53].

### The role of heat-stress and chronic dehydration

In addition to causing prolonged and extreme droughts and flooding, climate change has led to excessive heat exposure, dehydration, heat exhaustion, heat stroke, increased renal stone formation, and exacerbation of pre-existing chronic disease. Over the last century, global mean temperature has risen by approximately one degree [80], but has been associated with increased frequency of unpredictable weather, melting polar ice-caps, rising sea levels, severe flooding, heat waves and human ill health [81]. However, a direct connection between heat stress, local environmental changes secondary to global warming, potable water shortages, and behavioural and dietary sugar changes with CKDmfo has not been studied in-depth or confirmed [52].

Due to heavy exertion, lack of shade during work, a prolonged work hours during the day with little breaks, strenuous working conditions, and drinking insufficient water during work, outdoor workers are particularly at risk for heat stress and dehydration [82–85]. Nevertheless, the outdoor workers (mostly expatriates) who work long-hours getting daily exposure to scotching sun in middle-eastern countries, do not seems to develop CKDu-like syndrome.

Experimental studies in mice have demonstrated, recurrent daily heat exposure and dehydration causing chronic renal tubulo-interstitial disease with fibrosis and inflammation [86], similar to that observed in CKDmfo [81]. However, whether this is due to chronic rhabdomyolysis, elevated serum uric acid levels, other metabolic derangements, a combination of these factors, or something else, is unknown. Moreover, it is unclear whether this model is applicable to humans.

It is plausible that the toxicity of exposure to certain nephrotoxins (released to the environment as a consequence of having unsustainable development practices) can be additive in the presence of heat stress and chronic recurrent dehydration. Nevertheless, the rise of global temperature by one degree [81], causing CKDmfo seems highly unlikely.

For example, outdoor workers in the Middle-East, where the average temperate during summer exceeds 10°C than in the highest temperate exposure by the agricultural workers in CKDmfo affected regions, do not seem to develop CKDmfo/CKDu-like disease. Moreover, due to mechanised practices, the intensity of manual labour associated with rice- as well-as corn- and sugarcane-farming practices are less now, compared to several decades ago. For example, in south east Asia, there is a reduction of the number of strenuous working days annually, in paddy fields from an average of 90 to 42 (i.e., over a 50 % reduction of hard labour).

### Geographical distribution of CKD in the world

Most of the CKDuo-affected countries have hundreds of years of traditional, sustainable agricultural practices. As populations around the world increased, to increase yields, farmers began to abandon traditional cultivating methods when synthetic agrochemicals were introduced in the early 1960s [36, 87]. This led to improved food security in many emerging economies and in developing countries. Whether this Green Revolution has anything to do with the CKDmfo/CKDu is yet to be determined.

Organic farming as shown above, has its own issues. Recent data suggest that the use of organic farming is likely to decrease the excessive use of phosphates and nitrates which in turn reduces the leaching into the water system in comparison with the use of chemical fertilisers in agro-fields [88, 89], though not everyone agrees about this [90].

**Fig. 4:**
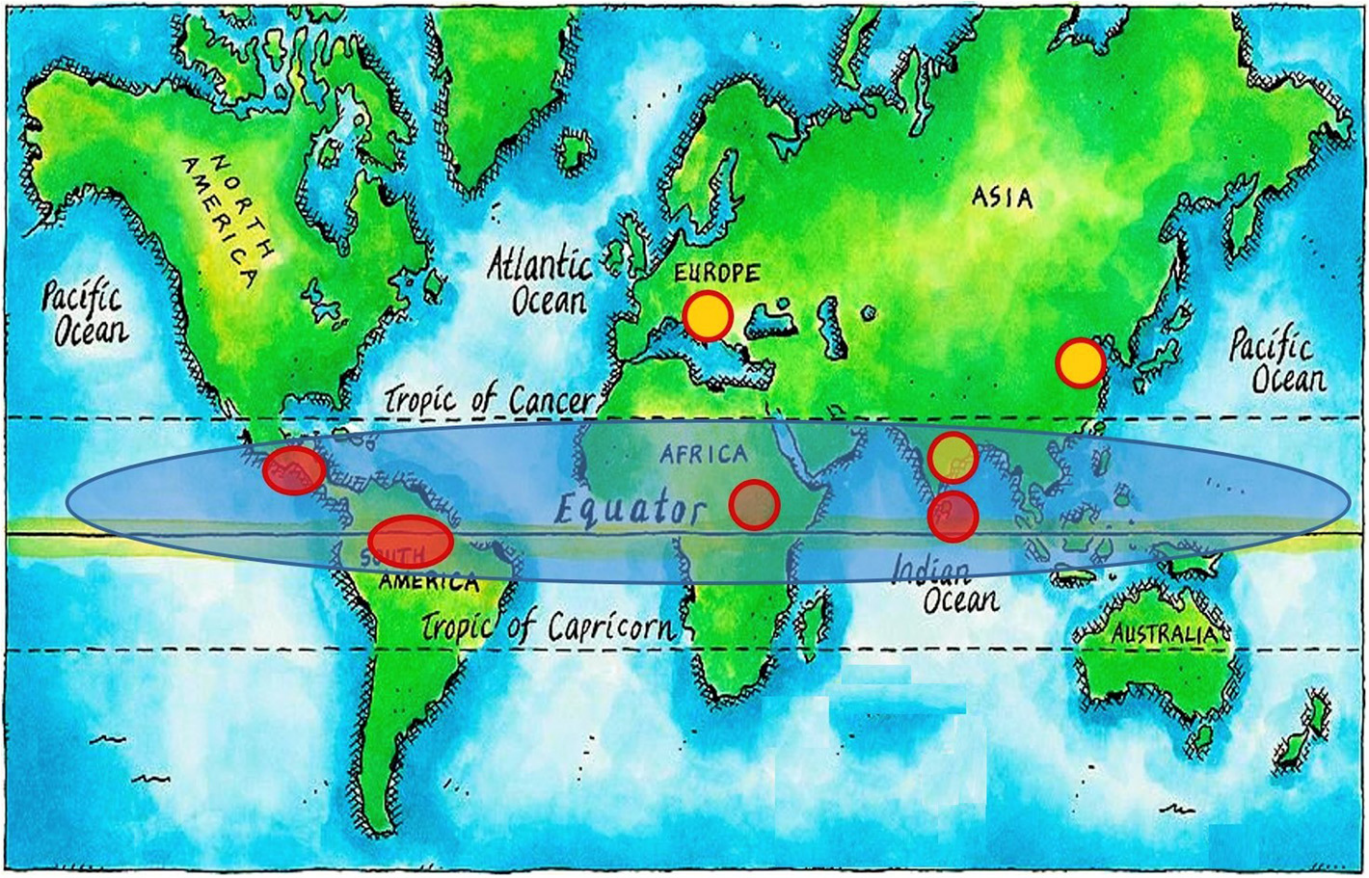
Global distribution of CKDu/CKDmfo. All affected countries are located close to the equator and have agriculture-based economies. Red circles indicate that the causes for CKDu/CKDmfo are unknown; yellow circles indicate countries in which some of the potential causes are understood. Some commonalities, especially the proximity to the equator of affected countries, are highlighted. The map is from the public domain, modified to indicate locations

Worldwide, there are several commonalities in the CKDmfo/CKDuo-affected countries, including a drought-stricken climate and proximity to the equator (Bangladesh, Sri Lanka, southern China, India, South America, and certain eastern European Balkan countries) (Fig. 4). Most of the affected nations are tropical and have developing or emerging economies that are predominantly agriculture based. It is noteworthy that all of these countries receive aid and loans from the World Bank, the International Monetary Fund, and industrialized nations.

Countries that are receiving such aid packages or loans are expected and have been obligated to follow some policies and procedures that may be imperil and not appropriate for these countries, citizens, or their economy. How these undesirable, imposed practices affecting the economy (in fact, a poverty-trap) of recipient countries, healthcare and chronic health conditions have not been addressed. All affected communities are relatively less wealthy and have insufficient access to clean water, sanitation, education, and modern healthcare [46, 91].

Despite claims by certain people, the geographical distribution of CKDmfo and the hardness of the water in all affected countries, including Sri Lanka, do not coincide, suggesting that the latter does not contribute to the development of chronic kidney failure [46]. In fact, some areas in Sri Lanka, such as the Jaffna Peninsula and the Puttalam and Ampara districts have two to three times greater water hardness and higher electrical conductivity and salinity in their drinking water than that in the CKDmfo-affected regions; NCP and the Kurunagala and Badulla districts [52], [48].

## Discussion

Because of the unique chemistry of the water molecule, water is easy to pollute; it has the ability to dissolve almost any substance [4, 92]. Therefore, some degree of water pollution is inevitable. Nevertheless, proactive environmental and public health interventions are essential to prevent pollution and ill health [93]. Public health interventions are usually intended to prevent future catastrophes [94]. However, certain actions can be taken now, which do not depend on decisions on how to safeguard people in the future.

Multi-country ecological studies have revealed that there are key environmental factors that contributes to chronic ill health and diseases including cancer, is adverse environmental issues and pollution, not genetics [95–97] Therefore, a cleaner environment and healthier lifestyles (both of which can be proactively improve) need to be emphasised. Such efforts would reduce the escalating incidences of cancer and almost all other non-communicable diseases, improve the health status and the economy of the nation, and the burden on healthcare budgets.

Imposing a strict quality control program on locally produced and imported agrochemicals and regulating the release of fertilisers to farmers based on soil testing are likely to decrease the agrochemical usage and overuse-associated, environmental pollution. As the discussion of the proposed causes of CKDmfo has shown, the search for agreement on the cause or causes of these diseases is likely to continue for many more years, thus, focusing on the available resources and evidence for prevention is the best way forward for eradication of communicable as well as non-communicable diseases. In this regards, CKDmfo is not an exception.

To curtail chronic disease associated escalating healthcare costs, countries must address the root causes of these illnesses. Adequate environmental protection and prevention of diseases should be part of that effort. Such preventative approaches lead to reduction in morbidity and mortality and are often more cost-effective than spending taxpayer money on expanding hospitals, purchasing expensive medical equipment, organ transplants, and treating escalating non-communicable diseases.

With agrochemicals being one of the key sources of environmental pollution, agribusinesses have a corporate social responsibility at least to invest in educating farmers on the proper use of such chemicals and provide subsidised or free protective gear to them [49]. On the other hand, farmers must be accountable for the proper use of agrochemicals and protecting themselves when handling these chemicals, and for taking care of the environment and all living beings around them.

Thus, it is paramount that agriculture-related personals are educated on various means of preventing environmental pollution, the protection of themselves and others, and the reasonable and responsible use and disposal of agrochemicals and containers. For such a program to be successful, approaches should be collaborative, not purely punitive, and should offer incentives.

Available data do not support any of the postulated agents, chemicals, heavy metals, fluoride, salinity/ionicity, or individual agrochemical components, such as phosphate or glyphosate, as causative factors for CKDmfo. Whereas, water and food pollution together with prolonged drought associated issues and conditions, and harmful personal behaviour are plausible causes of this deadly disease. Because of the plausibility of multiple factors playing a role in the genesis of this disease, the appropriate terminology is CKDmfo, rather than CKDu.

However, as the CKDmfo name implies, a combination of these factors (including unknown nephrotoxins) together with harmful behaviour and chronic dehydration are likely to cause this disease. Among the recent epidemics, CKDmfo may be one of the first diseases manifesting due to global warming and unsustainable developmental practices. Regardless of the cause, prevention and continued investigation of the possible causes of this disease must be continue; these activities should provide a path to eradication of the disease.

Few social support structures exist in the NCP and in most villages in developing countries. Foundations should be established to protect people from day-to-day life issues and unexpected events and to provide access to emergency care, such as in the aftermath of hurricanes, floods, tsunamis, and so forth. Properly taking care of a those who are affected and taking firm and effective steps to prevent the disease are prime responsibilities of health ministries and departments of health.  Having a tested emergency preparedness plan that can launch immediate programs of support in the event of emergencies at the community and regional levels is crucial to preventing deaths and containing communicable diseases.

With reference to the current calamity of CKDmfo, it is essential to provide clean drinking water and safe sanitary facilities as a priority for all affected regions and the entire country. Chronic disease management programs in the country need to coalesce and synergise to be cost-effective [98]. Despite the existence of CKDmfo for more than two decades, access to safe, clean water has been provided to less than 10 % of the needy population in the districts affected by this disease in Sri Lanka and other affected countries [52].

Considering that most shallow wells are contaminated (perhaps with unknown nephrotoxins) in CKDmfo-affected areas, and streams and reservoirs are increasingly getting  polluted, facilities should be developed urgently at least on a provincial basis to test for water quality and food contamination.

In the interim, potable water can be successfully and cost-effectively provided through reverse osmosis units, until centrally purified, safe, pipe-borne water supplies are made available in these regions. It is scandalous that in both rich and poor countries, people in rural villages are not provided with safe running water, while the government provides excellent facilities to wealthy and city populations. Gross inequalities in the supply of drinking water, lack of safe sanitation, and correctable healthcare disparities are not ethically acceptable in modern society.

## Conclusion [Box offering]

### What is already known on this subject?

Environmental pollution leads to various types of human ill health and occupationally acquired diseases. Human ill health can be caused by acidification of inland water bodies and the ocean; acid rain; atmospheric carbon dioxide and ozone issues; and water, air, and soil contamination, and so forth. Such pollutions lead to increased incidence of non-communicable diseases, such as obesity, hormone disruptions, and diabetes; preventable injuries; cerebral, liver, and renal diseases; asthma and allergies; chronic lung disease; and occupational diseases, such as CKDmfo. The mechanisms involved in environmental-pollution–causing diseases are not fully understood, but prevention is the path to cure.

### What this review adds?

Environmental protection should go hand in hand with the implementation of environmental laws to minimise water, air, and environmental pollution. The conditions are made worse by the proliferation of unregulated polluting industries that disregard environmental safeguards and laws, and people adopting lifestyles that are harmful not only to themselves but also to others and the environment. Correction of such measures will decrease several public health threats, especially non-communicable diseases. The continuation of these factors at current rates will lead to future calamities. Nevertheless, despite the harm already done to the environment and human health, the problems can be reversed and diseases can be prevented, if corrective proactive actions are taken now. To find sustainable solutions, governments should be responsible for water and food security and accountable for the environment and the public’s health and overall safety and security.
